# Ophthalmology

**Published:** 1876-09

**Authors:** 


### III. Ophthalmology.

1. *Ophthalmoscopy in Commotion and Contusion of the Brain.* Bouchut. (*Gaz. des Hôp.*, 123, 1875; *Centralbl. f. Chir.*, 12, 1876.)

Bouchut considers the examination of the fundus oculi with the ophthalmoscope as the surest diagnostic means by which to discriminate between the simple commotion and the graver or traumatic lesions of the brain. If there be commotion only, the fundus shows a normal appearance; but in the case of contusion of the brain, with or without any consecutive inflammation, and also in cases of a serous or hæmorrhagic effusion into the cranial cavity the signs of a disturbance of the circulation in the opthalmic veins will be found in the optic disc. This appears swollen, uniformly red and sometimes more vascular; its outlines are ill-defined, because the serous infiltration which extends from the papilla to the neighboring portion of the retina, renders the margin of the disc rather indistinct. On these signs of the "engorged papilla" Bouchut could make a sure differential diagnosis in four cases in which the clinical symptoms were very unsatisfactory, and left the diagnosis very doubtful.

2. *Affections of the Eye due to Diabetes Mellitus.* PROF. TH. LEBER. (*Arch. f. Ophth.* xxi., 3).

It is a well known fact that the crystalline lens sometimes becomes opaque (cataractous) in the course of diabetes mellitus; but this affection of the lens is not the only pathological change which occurs in the eye, or which could account for the dimness of sight the diabetic patients so often complain of. By a careful collection of the literary material, and by an analysis of a number of cases which fell under his own observation, Prof. L. is enabled to enumerate the following affections of the eye as occasional complications of diabetes mellitus, to-wit: 1, Hæmorrhage into the retina and vitreous humor; retinitis apoplectica, with or without degeneration of the retina; 2, amblyopia without any visible pathological changes in the fundus oculi; 3, atrophy of the optic nerve; 4, paralysis of the accommodation and sphincter pupillæ; 5, paralysis of ocular muscles. And it is interesting to learn that in several cases the patient's first complaint was about the sight, while he appeared yet to be in good health; the diabetes was detected, so to speak, through the disturbance it has caused in the functions of the eye. Prof. Leber therefore makes it his rule to test the urine of the patient for sugar, carefully and repeatedly, in every case of amblyopia without any satisfactory visible changes in the fundus.

3. *Neuro-retinitis caused by Lightning—Consecutive Blindness.* BRIERE (*du Havre.*) (*Gazette des Hôpit.*)

A young girl, aged 11 years, in excellent health previously, was caught in a violent thunder-storm while returning to her home from school. She did not look at the "zig-zags" of the lightning, as so many do imprudently, but cast her eyes toward the ground. The soil was, however, dry and blanched from the summer's sun of the previous days, and strongly reflected the light from the houses.

The girl had great difficulty in reaching her home in consequence of dizziness, which persisted during the evening. She slept well that night, but, on awaking, was almost blind, being unable to distinguish in the house anything more than the form of persons and the largest objects. In twenty-four hours the blindness was complete, there was only a feeble luminous sensation, and after waiting a fortnight the child was brought to B. The physiognomy and external aspect indicated the sequence of a profound affection of the ocular globes.

The pupils were widely dilated, and no longer sensible to the action of light and obscurity. The ophthalmoscope showed well-marked neuro-retinitis of both sides — choked papillæ, retinal œdema and venous hyperæmia. She could scarcely decide whether a lighted lamp was held in front of her. Minute doses of calomel, a leech daily behind each ear, synapisms on the nucha, and a dark chamber were ordered. Under this treatment there was moderate improvement for a time, so that the child could almost recognize its mother; but the idea became prevalent in the neighborhood that the patient was under the spell of witchcraft, and all scientific treatment was speedily exchanged for the ridiculous practices of superstition. Some months later B. heard that the little patient was totally blind.

An interesting point in this case was the fact that, though passing through a thunder storm, the child was so far distant from the electric explosions as to *hear no thunder*. There was consequently no injury by shock, but solely by intense reflected light.

